# Targets and Sources of Oral Corrective Feedback in English as a Foreign Language Classrooms: Are Students' and Teachers' Beliefs Aligned?

**DOI:** 10.3389/fpsyg.2021.697160

**Published:** 2021-06-25

**Authors:** Xuan Van Ha, Loc Tan Nguyen

**Affiliations:** ^1^Department of Foreign Languages, Ha Tinh University, Ha Tinh, Vietnam; ^2^School of Foreign Languages, University of Economics Ho Chi Minh City, Ho Chi Minh City, Vietnam

**Keywords:** oral corrective feedback, teacher beliefs, learner beliefs, feedback targets, feedback providers, Vietnamese EFL context

## Abstract

Recent decades have witnessed extensive research focusing on oral corrective feedback (CF), a key aspect of English as a second/foreign language (ESL/EFL) learning and teaching, but relatively little research has examined the relationship between learner and teacher beliefs about CF. The study reported in this article investigated the relationship between teacher and learner beliefs regarding the optimal targets and sources of CF in Vietnamese secondary EFL contexts. Data which were collected at four Vietnamese public high schools included questionnaires completed by 250 students, interviews with 15 of them, and interviews with 24 teachers. The findings showed that the students were happy to receive CF to all types of errors, including less important errors such as those not influencing their communicative success. The teachers were generally more selective in their choices of error types, but they sometimes faced some tensions between their overall teaching objective and the students' practical needs to learn the material that would be tested in subsequent exams. Regarding CF sources, the students preferred teacher correction to self-correction or peer correction although they believed that self-correction was effective for their learning and wished their teachers would provide them with training on how to conduct peer correction and self-correction. The teachers also thought that it was part of their role to be the main CF providers to ensure the accuracy of classroom feedback. Some teachers were skeptical about their students' ability to provide peer CF. Pedagogical implications are discussed.

## Introduction

Beliefs are “propositions individuals consider to be true [. . .] which are often tacit, have a strong evaluative and affective component, provide a basis for action, and are resistant to change” (Borg, 2011, p. 370–371). Learner and teacher beliefs have been a topic of interest in language education research for quite some time. Teacher beliefs are important because they can influence their classroom behaviors, and understanding teacher beliefs can provide insights into their teaching practices (Kagan, 1992; Borg, 2003, 2015, 2017).

Similarly, learner beliefs play an important part in facilitating the process and the outcomes of their learning (Ellis, 2008). A match between learner and teacher beliefs can enhance learning efficacy, but a mismatch can have detrimental effects on the learning process and outcomes. According to Ellis (2008), teachers need to “make their own beliefs about language learning explicit, to find out about their students' beliefs, to help their students become aware of and to evaluate their own beliefs and to address any mismatch between their own and their students' belief systems” (p. 24). Increasing our understanding of teacher and learner beliefs helps inform teachers' pedagogical choices to enhance the effectiveness of teaching and learning. The relationship between learner and teacher beliefs about language learning and teaching has received extensive research attention, but research investigating the relationships in beliefs about oral corrective feedback is relatively limited.

Oral corrective feedback (CF), i.e., teacher or peer responses to learners' erroneous utterances, is a topic of interest for both second/foreign language (L2) teachers and researchers in second language acquisition (SLA). Extensive research has examined the value of CF, revealing that CF has a facilitative role in L2 learners' language development (Russell and Spada, 2006; Mackey and Goo, 2007; Li, 2010; Lyster and Saito, 2010; Lyster et al., 2013; Nassaji, 2016). Research investigating the frequency and patterns of CF has shown that CF occurred frequently in various L2 settings, with recasts being the most frequent type of CF but they did not elicit learner uptake as frequently as explicit corrections or prompts (e.g., elicitation, repetition, and clarification requests) (Lyster and Ranta, 1997; Sheen, 2004; Lyster and Mori, 2006; Brown, 2016; Nassaji and Kartchava, 2020; Wang and Li, 2020).

Of all areas of CF research, CF beliefs have received the least research attention (Akiyama, 2017; Ha and Murray, 2020; Ha et al., 2021). Moreover, CF beliefs have been investigated as part of larger projects examining beliefs about language teaching and learning and were mostly elicited via a few questions asking students' and/or teachers' views about the efficacy of CF (Schulz, 1996, 2001; Brown, 2009; Loewen et al., 2009; Nguyen and Newton, 2019). This research agenda has shown that students were more positive about the role and necessity of CF than teachers were (Davis, 2003; Brown, 2009; Roothooft and Breeze, 2016; Li, 2017; Kim and Mostafa, 2021). For their part, CF beliefs have been argued to be distinctive from beliefs about grammar teaching (Loewen et al., 2009; Li, 2017), which suggests that more research is needed to investigate CF beliefs extensively. In addition, CF beliefs have been found to be context-specific (Schulz, 2001; Loewen et al., 2009), but little is known about beliefs of students and teachers in secondary English as a foreign language (EFL) contexts, especially those in Asian countries, including Vietnam (Ha et al., 2021). The current study extends this line of inquiry by investigating the relationship between students' and teachers' beliefs about the targets and sources of CF in Vietnamese secondary EFL contexts.

## Literature Review

### Students' and Teachers' Beliefs About the Targets of Oral Corrective Feedback

Targets of CF, or decisions regarding which errors should be corrected, are of critical pedagogical concern. As mentioned above, most previous research examining students' and teachers' beliefs about CF belonged to larger projects investigating beliefs about language learning and teaching in general (Schulz, 1996, 2001; Brown, 2009; Loewen et al., 2009; Li, 2017). This body of research primarily focused on the beliefs of students and/or teachers about the role and necessity of CF. Literature on students' and teachers' beliefs specifically about targets of CF is limited.

From a learning perspective, several studies have investigated the targets of CF, revealing mixed findings. An early study by Oladejo (1993) found that English as a second language (ESL) students at both high school and university levels in Singapore preferred “comprehensive, not selective” errors to be corrected to enhance their language accuracy. This finding was mirrored in Katayama's (2007) study where most of the 249 Japanese undergraduate EFL students wanted all errors to be corrected. Similarly, in a recent study with Chinese undergraduate EFL students, Zhu and Wang (2019) revealed that the students wanted all types of errors to be corrected, including less important errors which did not hinder communication. Zhang and Rahimi (2014) looked at Iranian undergraduate students' CF beliefs and anxiety levels. They found that the students valued the errors influencing communication the most, followed by frequent errors. Similarly, advanced ESL learners in the US felt that errors occurring most frequently in their speaking should be prioritized (Lee, 2013).

Regarding teachers, literature on their beliefs concerning CF targets is also limited. In his study involving 55 pre-service EFL teachers in Spain, Agudo (2014) found that only 33% of the teachers agreed with the idea of correcting all grammar errors, and 63% of the teachers thought that only some errors should be corrected to avoid discouraging students. In Jean and Simard's (2011) study with ESL teachers and French as a second language teachers (FSL) in Canada, only eight out of the 26 FSL teachers and three out of the 19 ESL teachers believed that grammar errors should be corrected all of the time. Fifty-four percent of the FSL and 68% of the ESL teachers agreed that only errors impeding communication should be corrected. The idea of correcting only errors related to the lesson foci received the endorsement of 46% of the FSL and 52% of the ESL teachers. This available literature illustrates that neither of the ideas of (1) correcting all errors, (2) correcting only errors influencing communication, or (3) correcting only errors related to the lesson focus, received strong support from teachers. As argued by Li (2017), the extreme statements (e.g., including such words as *all, only, every*) in those studies may have influenced the ratings by the participants.

Overall, the limited literature on students' and teachers' beliefs concerning CF targets shows mixed findings, suggesting that more studies are needed to yield meaningful conclusions regarding students' and teachers' preferences for CF targets.

### Students' and Teachers' Beliefs About the Sources of Oral Corrective Feedback

CF sources, or who should be the feedback providers, are a critical concern of both research and pedagogy (Ellis, 2009, 2017). Generally, studies on CF sources in different contexts showed discrepancies in their findings.

Several studies have investigated students' beliefs regarding CF sources. Schulz (1996, 2001), for example, found that only about 15% of the Columbian undergraduate EFL students and 13% of the US foreign language students preferred to be corrected by their peers in small group work. Partly consistent with Schulz's research, Agudo (2015) found that only 42% of Spanish undergraduate EFL students approved of receiving feedback from their peers in small group work. By contrast, 63% of the Japanese undergraduate students in Katayama's (2007) study wanted their peers to correct their errors in group work. In a quasi-experimental study, Sato (2013) found that Japanese undergraduate EFL students felt positive about the idea of having their classmates point out their errors both before and after the training, but they were less confident in giving feedback to their peers. This positive attitude to peer correction was also found with Chinese undergraduate EFL students in Zhu and Wang's (2019) study. This existing limited literature reveals that students were generally not positive about receiving CF from peers in small group work.

From a teaching perspective, recent research has shown that teachers shared a common belief about who should be the main CF providers within the language classroom. For instance, a majority of the 55 student teachers who participated in Agudo's (2014) study did not support the idea that peer correction is more effective than teacher correction, nor that peer correction causes less learner anxiety than teacher correction. Most of the student teachers (78%) highly valued the idea of teacher prompting for student self-correction. In a qualitative study with 15 university EFL teachers in Turkey, Yüksel et al. (2021) found that the teachers estimated that teachers should correct about 56% of students' errors, followed by self-correction (29%) and peer correction (15%). Similarly, Bao (2019) found that six out of the eight Chinese L2 teachers in his study thought that teachers should be the main CF providers.

In short, literature on CF targets and sources is limited and has produced inconsistent findings. Given the important role of CF in facilitating L2 learners' language development (Li, 2010; Lyster and Saito, 2010; Lyster et al., 2013; Nassaji, 2016), it appears timely to undertake more narrowly focused research in order to investigate CF beliefs exclusively. Moreover, to date, this research agenda has focused primarily on adult learners whereas beliefs about CF have been found to be influenced by contexts (Schulz, 2001; Loewen et al., 2009). Little is known about the beliefs of students and teachers regarding CF in such contexts as public secondary schools in Asia which involve a large population of EFL teachers and learners. More studies are, therefore, needed in a broader range of contexts to draw more meaningful conclusions which in turn can inform a research-based pedagogy within the language classroom. This study is timely in that it attempts to provide a missing piece for the larger puzzle by examining the relationship between students' and teachers' beliefs about the targets and sources of CF in Vietnamese secondary EFL contexts. It seeks to answer the following research questions:

1. What are Vietnamese EFL students' and teachers' beliefs about the targets of oral corrective feedback?
2. What are Vietnamese EFL students' and teachers' beliefs about the sources of oral corrective feedback?
3. Is there any (in)congruence between the students' and teachers' beliefs about the targets and sources of oral corrective feedback?

## Methods

This study adopts a mixed-methods research design (Tashakkori and Teddlie, 2010; Creswell and Clark, 2017) to explore the relationship between Vietnamese EFL students' and teachers' beliefs about the targets and sources of CF. Mixed-methods approaches can maximize the strengths and minimize the weaknesses of qualitative or quantitative data alone to advance the understanding of the phenomena being examined (Tashakkori and Teddlie, 2010; Riazi and Candlin, 2014; Creswell and Clark, 2017). In addition, given that students' and teachers' beliefs about CF are complex constructs (Basturkmen et al., 2004; Basturkmen, 2012), statistical results may not be adequate. Instead, more than one method of data collection and analysis is needed to yield more in-depth elaborations, explanations, and interpretations of the findings. The data comprised semi-structured interviews with 24 teachers, questionnaires with 250 students, and follow-up semi-structured interviews with 15 students (who completed the questionnaire). Before the data collection process started, ethical approval had been granted and all consent from the participants had been obtained.

### Contexts

The study was conducted at four public high schools (grades 10–12) in a central province of Vietnam. Within Vietnamese secondary education, English is a compulsory foreign language subject taught over three 45-min lessons per week. Great efforts have been made to improve the quality of English teaching and learning, and the expected outcome for high school students is a preliminary level of English proficiency (equivalent to level B1 of the Common European Framework of Reference for Languages). In reality, however, the teaching and learning of English is highly exam-oriented (Ha and Murray, 2021; Ha et al., 2021). In other words, teachers typically focus on helping students pass exams which mainly test learners' knowledge of vocabulary and grammar. The classroom is the main venue where students can use English for interaction. Some students engage in extra English lessons after class either at their school, at private language centers, or their teachers' houses.

### Participants

The participants were recruited using a convenience sampling method (Dörnyei, 2007). Research information and consent forms were delivered to students by their teachers. The students had time to read the information and their consent form at home before deciding to take part in the study. They then signed the consent form if they agreed to participate and gave it back to the teachers. The teachers with their students' signed consent forms contacted the first author to return them. The first 250 students returning their signed consent forms were selected to participate in the study. The student participants included 98 males and 152 females, aged between 15 and 17. By the end of the questionnaire, students were asked if they volunteered to take part in a follow-up interview. For logistical reasons, the first 15 volunteers, including seven males and eight females, were selected for the interview. The students' English proficiency ranged between elementary and intermediate although they had all started learning the language at Grade 6 or earlier.

The teacher participants were 24 EFL teachers with teaching experience ranging from 10 to 21 years (mean = 15.8 years). There were 23 females and one male, reflecting the unbalanced distribution of the foreign language teaching workforce in Vietnam. The teachers all had a bachelor's degree in teaching EFL. They had participated in various professional development activities but reported that they had never been involved in any discussion about CF theories or strategies. All the participants took part in the study on a voluntary basis, and pseudonyms (Teacher 1–Teacher 24; Student 1–Student 15) were used to ensure confidentiality.

### Instruments

The instruments for data collection included a questionnaire for students and interview protocols for students and teachers. The current study is part of a larger research project in which these instruments were developed.

The protocol for the teachers' semi-structured interviews was developed based on the comprehensive review of recent CF literature (e.g., Lyster et al., 2013; Nassaji, 2016; Li, 2017; Ha and Murray, 2020). It was designed to elicit teachers' beliefs about various aspects of CF. Within the scope of the present study, only data regarding beliefs about the targets and sources of CF were used.

The questionnaire for student participants was developed based on the synthesis of the literature about students' CF beliefs (e.g., Schulz, 1996; Loewen et al., 2009; Kartchava and Ammar, 2014) following guidelines on questionnaire construction (Dörnyei and Taguchi, 2009). Rigorous procedures of validation and piloting had been applied before the data were collected. All the questionnaire items were original. Initially, the questionnaire was developed in English by the first author and his colleagues who were involved in the larger study. All the items were then polished and revised through several meetings and discussions, and the questionnaire was translated into Vietnamese by the first author in consultation with two bilingual colleagues (Vietnamese and English) for accuracy and subtlety in translation.

To validate the content of the questionnaire, three teachers and five students from one of the two schools where the pilot study was conducted were invited to participate in the process. The validation was conducted with the teachers before it was trialed with the students. They were requested to read all the items thoroughly and complete the questionnaire in the presence of the first author. They were also encouraged to exchange ideas with the author regarding both the wordings and content of all the items. Based on the teachers' and students' comments, some amendments to several items were made. The pilot study was then carried out with 100 students at two high schools. They were not the student participants in the main study but were comparable in terms of age, English proficiency, and learning contexts. Analysis of the pilot study revealed several flawed items that were subsequently excluded for better scale reliability. Satisfactory reliability was achieved (α = 0.83), and the time range for the questionnaire completion was estimated (15–23 min).

The final version of the questionnaire consisted of two parts, with part one eliciting students' demographic background and part two focusing on students' CF beliefs. The second part comprised 47 Likert scale items and one ranking item eliciting students' beliefs about various aspects of CF. However, only items probing students' beliefs about the targets and sources of CF were reported in the present article. To avoid any difficulties in language on the part of the participants, the questionnaire was administered in Vietnamese, the participants' first language.

The development of the interview protocols for students was based on both the synthesis of the CF literature and the preliminary analysis of the questionnaire data. The interview was designed to elaborate on the quantitative findings of the questionnaire. The interview questions were initially developed in English and then translated into Vietnamese by the first author. The translation was cross-checked for accuracy by two colleagues who are bilingual in Vietnamese and English. The interviews were conducted in Vietnamese, the shared first language between the participants and the researchers.

### Data Collection and Analysis

The data collection started with teachers' interviews. Each teacher was interviewed individually at his/her school common staff room, and each interview lasted between 63 and 78 min. The paper-based questionnaires were delivered to 250 students to complete at their convenience. After one week, 247 completed questionnaires were returned. However, 11 of them were incomplete and were removed from the data set, leaving 236 questionnaires for data analysis. Students' interviews took place three weeks after the questionnaires had been completed. Each student was interviewed individually in a common staff room. Each interview lasted for approximately 22 min. All the students' and teachers' interviews were audio-recorded for data transcription and analysis.

Descriptive statistics were adopted to analyze the questionnaire data with the support of SPSS software (version 26). Within the scope of the current study, no factor analysis, nor inferential statistical analysis was adopted. The Cronbach's alpha value, in the present study, for the whole questionnaire was 0.85, and the figures for the CF target group and the CF source group were 0.714 and 0.703 respectively, indicating that the internal consistency for the instruments was acceptable (Dörnyei and Taguchi, 2009; Pallant, 2016).

The interview data were submitted to NVivo software (version 11) for thematic analysis (Braun and Clarke, 2006). Analysis of the students' interviews was conducted separately from that of the teachers' interviews. To explore the relationships between the students' and teachers' beliefs, findings from the teachers' interview data were compared and contrasted with those derived from the questionnaire data and the students' interview data. The findings from the students' interview data were used to explain, interpret, and contextualize the findings from the questionnaire data. The procedure of qualitative data analysis is as follows.

Firstly, the interview recordings were fully transcribed verbatim in Vietnamese by the first author. Only quotes used in this paper were translated into English and were cross-checked by the second author for accuracy in translation. Secondly, all the transcripts were read many times for a thorough understanding of the data. The phrases or sentences with similar meanings were grouped into categories. Finally, these codes were revised and refined to avoid overlap and redundancy and to develop themes. The emerging themes will now be reported and discussed.

## Findings

### Targets of Oral Corrective Feedback

The students' beliefs about the targets of CF were elicited through seven items. As can be seen in Table 1, all items had a mean score of over 4.0 out of 5.0, indicating that the students were positive about receiving CF to all types of errors. The three types of errors that received the highest ratings were errors influencing communication (Q29), frequent errors (Q31), and errors related to the lesson focus (Q32). Interestingly, the errors seen as less important, such as errors which are not likely to influence communication (Q30), less frequent errors (Q33), and errors not related to the lesson focus (Q34), also received high levels of approval.

**Table 1:** Students' beliefs about targets of oral corrective feedback.

	***N***	**Min**	**Max**	**Mean**	**SD**
Q28 All errors should be corrected.	236	1	5	4.00	0.843
Q29 The errors that impede communication are the most important and worth correcting.	236	1	5	4.08	0.839
Q30 Some errors do not impede communication, but it is necessary to correct them.	236	1	5	4.01	0.777
Q31 The errors that students make frequently are the most important and worth correcting.	236	1	5	4.08	0.864
Q32 The errors related to the focus of the lesson are the most important and worth correcting.	236	1	5	4.07	0.804
Q33 Some errors are not common in the class, but when they occur, they need to be corrected.	236	1	5	4.03	0.714
Q34 Some errors are not related to the focus of the lesson, but they need to be corrected.	236	1	5	4.01	0.747

These quantitative findings were supported by the qualitative findings from the interview data. All of the interviewed students stated that all kinds of errors should be corrected because they would like to improve their language accuracy. They said that accuracy is the most important for them, not only for communication but also for different kinds of exams. When asked which types of errors should be prioritized, most of the students reported that frequent errors and errors related to the focus of the lesson were the most important and worthy of correction, as evidenced in the following comments:

I think errors related to the lesson focus are the most necessary to be corrected because we need to understand the lesson. (Student 3)

For me, the main thing of speaking is to make people understand, so errors that are likely to hinder communication are the most worthy of correction. Otherwise, our communication will fail. (Student 12)

Interestingly, when asked if less important errors should be corrected, 12 of the students said that even though some errors may not influence communication or were not the focus of the lesson, they should be corrected. They reasoned that they learned English for different purposes but exams were of the highest priority. For example, Student 6 stated, “minor errors such as misuse of plurals or singulars, and subject-verb agreements should be corrected too. They may not influence communication at that time but are important for exams.”

Analysis of the interviews with the teachers showed that there was some congruence and some incongruence between the beliefs of the teachers and students regarding CF targets. All of the 24 teachers considered that although CF is beneficial for students' learning and necessary in L2 classrooms, it should be provided selectively due to practical reasons such as time, class size, and an excessive number of learners' errors. Some teachers also believed that correcting one student too frequently may bring about negative effects on his or her well-being and emotional state. They mentioned that whether to correct or not depends on students' proficiency and individual differences. On the question of which errors worthy of correction the most, the teachers considered that errors related to the focus of the lesson should be given higher priority. This aligns with their students' stated beliefs as reported above. Following is a typical comment from Teacher 2:

All kinds of errors should be corrected, but many of them need to be ignored because we can't correct them all. It depends on the lesson. For example, in a grammar lesson, I will focus mostly on the errors related to the language structure of the lesson. In a speaking lesson, I will focus on pronunciation errors.

Eight of the teachers considered the CF targets in relation to the timing of CF. They believed that errors influencing communication should be corrected immediately, while some error types could be delayed. According to the teachers, frequent errors which occurred with many students at the same time can be delayed until the end of a speaking activity because they needed to spend more time and effort on explaining to the students carefully about the rules.

Ten of the teachers stated that they need to consider a number of factors in their decisions about which errors should be corrected and which could be ignored or delayed. There were some tensions in the teachers' beliefs in this regard. On the one hand, they would like to help students develop overall speaking competence such as fluency, standardized pronunciation, or target-like intonation. On the other hand, the teachers believed they should prioritize preparing for students' exams which were in written forms testing students' knowledge of grammar and vocabulary. For example, Teacher 20 said:

Fluency and comprehensibility are important in students' speaking performance. However, we do not teach for communication only, but for exams as well. Exams will test students' knowledge about what they have learned in terms of vocabulary, sentence structures, prepositions, phrasal verbs, etc. So, we need to make sure students don't make these errors in their exams.

### Sources of Oral Corrective Feedback

Students' beliefs regarding CF sources were examined with 10 items. As shown in Table 2, the two items about the effectiveness of self-correction (Q21 and Q25) received high mean scores (mean = 3.99 and 3.97 respectively). Also, the item asking about students' wishes to be trained for self-correction and peer correction received the highest mean score (Q27, mean = 4.03). The item asking students' preference for teacher correction (Q19) received a higher level of approval (mean = 3.7) than those items asking about their preferences for teacher identification of error for self-correction (Q20, mean = 3.45) and teacher identification of error for peer correction (Q22, mean = 3.19). Interestingly, the item asking students' views about peer correction without teachers' pointing out errors (Q24) received the lowest level of approval (mean = 2.49). Also, the statement saying “self-correction or peer correction is more beneficial than teacher correction” (Q26) received a very low level of approval (mean = 2.73). These findings suggest that although students highly valued the effectiveness of self-correction and wished to be trained to do self-correction and peer correction, they also highly valued the role of teachers in pointing out errors and providing the correct answers. It seems that students were not confident in receiving CF from peers without any involvement of teachers.

**Table 2:** Students' beliefs about the sources of oral corrective feedback.

	***N***	**Min**	**Max**	**Mean**	**SD**
Q18 My teacher should be the one who gives me feedback on my errors.	236	1	5	3.25	1.051
Q19 My teacher should be the one who gives me the correct forms of my errors.	236	1	5	3.70	0.864
Q20 My teacher should point out my errors so that I can correct them by myself.	236	1	5	3.45	1.065
Q21 My teacher should encourage students' self-correction because it is helpful for them.	236	1	5	3.99	0.968
Q22 My teacher should point out my errors so that my classmate can correct them.	236	1	5	3.19	1.028
Q23 I want my classmate to point out my errors.	236	1	5	3.34	0.887
Q24 I want my classmate to correct my errors without my teacher's pointing them out.	236	1	5	2.49	0.887
Q25 If I correct my errors by myself, it will be useful for my learning.	236	1	5	3.97	1.025
Q26 Self-correction or peer correction is more beneficial than teacher correction.	236	1	5	2.73	1.127
Q27 I want my teacher to train me and my classmates to provide feedback to each other.	236	1	5	4.03	0.892

Explanations for students' preferences for CF sources were provided in the interviews. All the interviewed students said that they were comfortable with their teachers' corrections because teacher correction was a very frequent activity in their classes. Additionally, they thought that teacher CF could help them avoid similar errors in their speaking and subsequent exams. From the students' perspectives, teachers were the ones who could provide the best answers for their erroneous utterances. Twelve of the students commented that they would like to self-correct their errors following teachers' prompts because if they could self-repair, they would feel proud and their teachers might feel happy about them too. For example, Student 15 said “if I can correct myself after my teacher identifies my errors, it will help me to remember the language feature for long. More importantly, I feel very happy just like I have won something.” Regarding peer correction, the students expressed a general concern that they sometimes did not feel very comfortable receiving CF from their peers. Also, some peers may not be able to give accurate corrections to their errors. Interestingly, five of the students said that sometimes they were afraid of being judged by their peers. For example, Student 5 said, “some people may look down on their friends if they make simple errors.”

Analysis of the teacher interviews showed that there were some similarities and some differences between the students' and teachers' beliefs. Fifteen of the 24 interviewed teachers were positive about all sources of CF, commenting that either teacher correction, self-correction, or peer correction was effective. They believed that teacher correction was the most common because it was quicker and easier to deliver and it could secure accurate corrections. The teachers elaborated that self-correction could help students retain the target language features better and longer, and peer correction was helpful for both the correctors and the receivers. In line with the students' beliefs, seven teachers believed that self-correction could give students a sense of pride, which could encourage them to participate more actively in classroom activities. However, half of the teachers did not believe in the ability of their students to do peer correction. For example, Teacher 8 said, “many students in my classes cannot perform peer correction because they are too weak.”

Taken together, the study found that both the teachers and students held strong beliefs in the importance of CF to learners' language development and that both groups of participants were more positive about teacher correction than peer correction. However, they did not share their views on what types of errors are worth correcting and who should be the main CF providers. While the teachers were selective in correcting their students' errors, the students would have preferred all their errors to be corrected.

## Discussion

The findings showed that the students were very positive about the importance and necessity of CF to all kinds of errors, including less important errors. It is not surprising that all the students in our study considered that important errors including those influencing communication, related to the lesson focus, or occurring frequently with them, were necessary to correct. What is more notable is the students' belief that less important errors were worthy of correction as well. This finding is in line with that in research in some Asian EFL contexts, such as studies of Katayama (2007) with Japanese undergraduate EFL students and Zhu and Wang (2019) with Chinese undergraduate EFL students. However, it is different from the finding of Lee's (2013) study which found that advanced ESL learners learning English for communication in the US prioritized CF for errors occurring frequently in their speaking.

The finding that the students in the present study felt that CF should target all error types may be due to the influence of the learning and teaching contexts (Loewen et al., 2009; Ha and Murray, 2020, 2021). The fact that exposing to frequent teachers' CF to all types of errors may have influenced the students' preferences for error types. According to the students' comments in the interviews, they had received CF very often and CF had been provided to all error types. Another explanation for this finding is the influence of the exam-oriented teaching and learning culture in Vietnam (Ha, 2017; Ha and Murray, 2020, 2021). As explained by the interviewed students, fluency in speaking was important, but they preferred to improve their language accuracy to score high in their subsequent exams. This is also evidenced in the students' stated beliefs about the priority for errors related to the lesson focus.

The teachers' beliefs about CF targets partially matched their students' beliefs. On the one hand, the teachers thought that CF targets should be more selective, believing that important errors, especially those related to the lesson focus, should be prioritized. The teachers cited practical constraints such as class size, time limit, and high frequency of errors for their selective correction. They were also concerned about students' well-being and emotional state, stating that too much correction may discourage students from participating in future activities. This concern has been reported in previous studies (Roothooft, 2014; Kamiya, 2016; Roothooft and Breeze, 2016; Li, 2017). On the other hand, rather than being influenced by concerns about students' affective responses to CF, the teachers in the present study showed some tensions between their overall objective of teaching, aiming to develop students' speaking competence in general, and fluency in particular, and the more practical objectives of the teaching and learning in their local contexts. Previous studies in teacher cognitions show that tensions and trade-offs between different sets of beliefs may lead to teachers' mismatches between their beliefs and practices (Phipps and Borg, 2009; Ha and Murray, 2020). It seems that, in the present study, the teachers' priority for helping students achieve good results in written exams which mainly tested students' knowledge of grammar and vocabulary exerts a stronger influence on their beliefs about CF targets than their aim to develop students' speaking fluency. This consideration resulted in the teachers' decisions on correcting less important and local errors which are unlikely to influence communication such as the use of articles (i.e., a, an, the) or subject-verb agreement.

Regarding students' preferences for the sources of CF, the students in this study preferred teacher correction rather than self-correction or peer correction. This finding aligns with that in previous studies (Schulz, 1996, 2001; Agudo, 2015). The students provided several reasons for their preferences in the interviews. Specifically, they felt more secure with teacher correction, believing that teacher correction ensures correctness, while peer correction may cause some face-threat concerns. Interestingly, although the students in this study preferred to receive feedback from teachers rather than peers, they highly valued the efficacy of self-correction and wished to be trained in self-correction and peer correction. As they explained in the interviews, some students felt proud after they could self-repair their erroneous utterances.

The teachers were positive about all three sources of CF, believing that teacher, self-correction or peer correction was useful. However, they stated that teachers should be the main source of CF because teacher CF was quick and easy to deliver. This belief was similar to that of the teachers in studies by Bao (2019) and Yüksel et al. (2021). What is notable in the current study findings is that although the teachers generally believed in the efficacy of self-correction and/or peer correction, one third of them were suspicious about their students' ability to provide CF to their peers. In these particular EFL settings, such suspicion may be attributed to the students' low level of English proficiency. Another explanation for this finding and the belief that teachers should be the main source of CF is the influence of the traditional educational role relationship between students and teachers in Vietnam. Within the context of Vietnamese EFL education, teachers are seen as experts and knowledge givers while students are considered as knowledge receivers (Ha, 2017; Ha and Murray, 2020), which may have resulted in the teachers' belief that teachers should be the main source of CF. This cultural value may also account for the finding that students preferred teacher correction rather than self- or peer correction because they believed in the expertise of their teachers.

## Limitations and Future Directions

Despite the contributions discussed above, the findings of the study should be interpreted with some caveats in mind. Firstly, the current study used a convenience sampling method, possibly attracting the most enthusiastic and outgoing students. Thus, it is necessary that future research should adopt a different sampling method to include a wide range of student participants who vary in their English proficiency, learning goals, motivation, and learning styles, and so on. Secondly, given that students' and teachers' beliefs have been found to be situated, complex, and dynamic (Leontjev, 2016; Akiyama, 2017), the one-shot questionnaires and interviews in this study may not have been able to capture the dynamics of CF beliefs. Future studies may therefore consider taking a longitudinal approach, for example, asking learners to keep a diary to track changes in their beliefs over time, or using a series of interviews at different points of time. It would also be interesting to see whether teachers' beliefs and practices regarding CF are sustained or whether they change over time.

## Conclusion and Pedagogical Implications

The current study represents an explanatory first step in understanding the relationship between EFL teachers' and students' beliefs about oral CF targets and sources. It investigated Vietnamese secondary EFL students' and teachers' stated beliefs about what errors should be corrected and who should provide the correction within the language classroom. The findings showed that the students were positive about CF to both important errors (e.g., errors influencing communication and those related to the lesson focus) and less important errors (e.g., errors not impeding communication). In contrast, the teachers were more selective in their CF choices, and they faced some tensions in making decisions on their CF targets in relation to their overall teaching objective and the practical needs of their students. This dissonance between the views of the teachers and students on which types of errors deserve more attention suggests that there is room for discussions between teachers and learners so that both can gain better understandings of the views of the other and modify practices and expectations accordingly.

The study has also shown that both the students and teachers expressed the belief that teachers should be the main CF providers, but were convinced that self-correction and peer correction were also effective. Some students were concerned about the possible face-threatening nature of peer correction, while some teachers expressed their skepticism about their students' ability to give peer feedback. The findings that the teachers were skeptical about their students' ability to provide peer correction and that the students highly valued the effectiveness of self-correction and expected their teachers to train them on its provision suggest that it would be of value for teachers to consult with their students regarding the benefits and strategies of self-correction and peer correction. For example, teachers could organize open discussions about students' beliefs regarding peer CF. Given that peer CF has been shown to be trainable (Sato, 2013), teachers should organize hands-on activities that focus on giving peer CF and create a supportive atmosphere in the classroom. In this way, teachers can create opportunities for students to be explicit about their CF beliefs, and to reflect on their CF practices while they receive CF from teachers and peers and while they give CF to their peers. This kind of reflection may increase the learner noticing and uptake of CF, ultimately enhancing the effectiveness of learning.

## Data Availability Statement

The original contributions presented in the study are included in the article/supplementary material, further inquiries can be directed to the corresponding author/s.

## Ethics Statement

The studies involving human participants were reviewed and approved by Human Sciences Ethics Subcommittee Macquarie University. The patients/participants provided their written informed consent to participate in this study.

## Author Contributions

XH contributed to the conception and design of the study, collected and analyzed the data, and wrote the manuscript. LN wrote sections of the manuscript. Both authors contributed to manuscript revision, read and approved the submitted version.

## Conflict of Interest

The authors declare that the research was conducted in the absence of any commercial or financial relationships that could be construed as a potential conflict of interest.
